# Prognostic value of a novel biomarker combining DNA ploidy and tumor burden score for initially resectable liver metastases from patients with colorectal cancer

**DOI:** 10.1186/s12935-021-02250-x

**Published:** 2021-10-23

**Authors:** Jianhong Peng, Weihao Li, Wenhua Fan, Rongxin Zhang, Xinyue Li, Binyi Xiao, Yuejin Dong, Desen Wan, Zhizhong Pan, Junzhong Lin, Xiaojun Wu

**Affiliations:** 1 Department of Colorectal Surgery, State Key Laboratory of Oncology in South China, Collaborative Innovation Center for Cancer Medicine, Sun Yat-sen University Cancer Center, Guangzhou, 510060 Guangdong P. R. China; 2 NingBo Meishan FTZ MBM Clinical Lab Co., Ltd, Ningbo, 315832 Zhejiang P. R. China

**Keywords:** Colorectal cancer, Liver metastases, DNA ploidy, Stroma fraction, Nucleotyping, Prognosis

## Abstract

**Background:**

Colorectal cancer liver metastases (CRLM) has not been identified as a unified disease entity due to the differences in the severity of metastatic disease and tumor aggressiveness. A screen for specific prognostic risk subgroups is urgently needed. The current study aimed to investigate the prognostic value of DNA ploidy, stroma fraction and nucleotyping of initially resectable liver metastases from patients with CRLM.

**Methods:**

One hundred thirty-nine consecutive patients with initially resectable CRLM who underwent curative liver resection from 2006 to 2018 at Sun Yat-sen University Cancer Center were selected for analysis. DNA ploidy, stroma fraction and nucleotyping of liver metastases were evaluated using automated digital imaging systems. Recurrence-free survival (RFS) and overall survival (OS) were analyzed using the Kaplan-Meier method and Cox regression models.

**Results:**

DNA ploidy was identified as an independent prognostic factor for RFS (HR, 2.082; 95% CI 1.053–4.115; *P* = 0.035) in the multivariate analysis, while stroma-tumor fraction and nucleotyping were not significant prognostic factors. A significant difference in 3-year RFS was observed among the low-, moderate- and high-risk groups stratified by a novel parameter combined with the tumor burden score (TBS) and DNA ploidy (72.5% vs. 63.2% vs. 37.3%, *P* = 0.007). The high-risk group who received adjuvant chemotherapy had a significantly better 3-year RFS rate than those without adjuvant chemotherapy (46.7% vs. 24.8%; *P* = 0.034).

**Conclusions:**

Our study showed that DNA ploidy of liver metastases is an independent prognostic factor for patients with initially resectable CRLM after liver resection. The combination of DNA ploidy and TBS may help to stratify patients into different recurrence risk groups and may guide postoperative treatment among the patients.

**Supplementary Information:**

The online version contains supplementary material available at 10.1186/s12935-021-02250-x.

## Introduction

Colorectal cancer (CRC) is the third most common cancer worldwide and one of the leading causes of cancer-related death [1, 2]. Distant metastasis remains a major cause of treatment failure and death for patients with CRC, and liver metastasis is the most common pattern, accounting for approximately 50% of cases [3, 4]. Although patients with initially resectable colorectal liver metastases (CRLM) achieve a 47.3%–50.2% five-year overall survival (OS) rate after liver resection, more than 80% of patients develop postoperative recurrence, which exceeds 50% within the first 2 years [5, 6]. To date, the benefits of postoperative treatment have not been definitively shown, and the choice of adjuvant chemotherapy for patients with initially resectable CRLM remains controversial [7, 8]. Therefore, the management of CRLM is challenging, and studies exploring novel clinicopathological characteristics to identify various prognostic subgroups with different risks of tumor recurrence and guide personalized treatment are urgently needed [9, 10].

In recent decades, several important clinicopathological factors have been consolidated into prognostic scoring systems for patients with CRLM receiving curative liver resection, such as the Nordlinger score [11] and the clinical risk score [12]. Recently, accumulating studies have reported that the “tumor burden score” (TBS) developed based on the tumor size and the number of liver metastases showed better prognostic discriminatory power than the clinical risk score for patients with CRLM [13, 14]. However, those scoring systems were only generated based on the gross level of the tumor, and the tumor cell structure level was not specifically considered. The combination of the characteristics of tumor growth and cell structure is expected to evaluate the risk of recurrence more accurately.

Several pathological parameters of the tumor cell structure have been shown to have prognostic value in CRC. Chromosomal instability (CIN), one of the major types of genomic instability recognized as an alternative mechanism of CRC, is present in approximately 65–70% of patients and is often inferred from DNA ploidy [15, 16]. Nondiploid DNA ploidy has also been suggested to promote or suppress CRC development and may even be associated with a poor prognosis of patients with CRC [17–20]. Stroma fraction was defined as the ratio of the area occupied by carcinoma cells to the total area occupied by stromal cells and carcinoma cells in hematoxylin and eosin (H&E)-stained tissue sections. Previous studies have reported that a high stroma fraction is associated with a poor prognosis for patients with CRC [21, 22]. Moreover, the combination of stroma fraction and DNA ploidy has been validated to reliably stratify subpopulations of patients with stage II/III CRC presenting with different risks of recurrence in several cohort [23, 24]. Chromatin organization, including the chromosome structure, position and number, may affect nucleotide polymorphisms and genome arrangement, regulating gene expression and changes during cell differentiation [25]. Nucleotyping, the application of machine learning image analysis methods to images that depict chromatin organization in cell nuclei, has been shown to be a pan-cancer prognostic factor [26]. To the best of our knowledge, although the prognostic value of DNA ploidy, stroma fraction, and nucleotyping in primary colon tumors has been extensively investigated, the results from liver metastases are relatively lacking.

The present study applied automated digital imaging systems to evaluate DNA ploidy, stroma fraction, and nucleotyping in liver metastases from patients with initially resectable CRLM. Accordingly, we aimed to (1) describe the characteristics of DNA ploidy, stroma fraction and nucleotyping in liver metastases, (2) investigate the prognostic value of the three pathological parameters in liver metastases from patients undergoing liver resection, and (3) identify novel parameters combined with the TBS to stratify patients into different risk groups.

## Materials and methods

### Patient population

We reviewed clinical data from 583 consecutive patients with initially resectable CRLM who underwent primary tumor and liver resection from April 2006 to October 2018 at Sun Yat-sen University Cancer Center. Patients included in the final analysis satisfied the following inclusion criteria: (1) histologically confirmed colorectal adenocarcinoma, (2) metastases limited to the liver, (3) no preoperative chemotherapy before liver resection, (4) radical resection of both the colorectal primary tumor and liver metastases, (5) at least a 3 month follow-up period after liver resection, (6) available formalin-fixed and paraffin-embedded (FFPE) samples of liver metastases, and (7) sufficient tumor tissue for detection. Informed consent for the use of the tissue samples was obtained from the patients before tumor resection. The study was approved by the Institutional Research Ethics Committee of Sun Yat-sen University Cancer Center (approval number: B2020-294-01).

### Tumor sampling

For DNA ploidy, stroma fraction and nucleotyping analyses, a pathologist selected a tumor block considered representative from each patient and delineated the entire epithelial tumor region. The three pathological parameters were detected at Ningbo Meishan FTZ MBM Clinical Lab Co., Ltd.

### DNA image cytometry

A 5-µm FFPE section was sliced, and the tumor region was defined by performing H&E staining. One 50-µm section containing more than 50% of the representative tumor tissue was sliced from the FFPE block where the tumor-rich area was marked. The nuclei of the tumor cells were released as previously reported [27]. A volume of 100 µl of the solution was centrifuged at 600 rpm for 5 min on a Cytospin to prepare a monolayer of nuclei on a slide. The monolayer slides were air dried and fixed overnight with 4% formaldehyde before Feulgen’s staining [28].

### Measurement of the DNA content

Images of the Feulgen-stained nuclei were captured with a DNA ploidy Working Station (Room 4, UK), as previously reported [28]. An image of the monolayer was captured using a high-resolution digital scanner (Aperio AT2, Leica, Germany), and the images of nuclei were automatically grabbed by PWS grabber software (Room4, UK) and grouped into different galleries of tumor nuclei, reference nuclei and discarded nuclei. DNA content histograms were generated from the integrated optical density (IOD) of the nuclei using PWS Classifier (Room 4, Kent, UK). Using lymphocyte nuclei as an internal reference, the DNA ploidy histograms were classified into four categories, diploid, aneuploid, tetraploid and polyploid, according to a previous report [27].

### Nuclear texture analysis

Nucleotyping, which is evaluated from the chromatin value, was automatically calculated using the method proposed in a previous study [26]. Each tumor sample was grouped using the PWS Classifier from the same set of images of tumor nuclei that was used to construct the DNA ploidy histogram. The chromatin configuration was evaluated by computing the entropy of pixel gray levels in each pixel of a nucleus. The frequency at which each pair of entropy and center gray level occur throughout a nucleus was stored in a two-way table, known as the gray level entropy matrix (GLEM). GLEMs stratified by nuclear area and subregion size were concatenated to form a four-dimensional expansion of the GLEM called GLEM4D. An adaptive machine learning algorithm was applied to quantify the association between each element of GLEM4D and the outcome of the patient. In the current study, these pretrained weights were directly applied to predict the outcome of a patient based on the GLEM4D representation of its tumor. This procedure was performed by multiplying each element of the patient’s GLEM4D with the corresponding weight computed and then summing the products. The result is a continuous value termed the chromatin value that describes the overall amount of chromatin state in a particular sample, and based on a previously established threshold of 0.044, the tumors were classified into chromatin homogeneous (CHO, ≥ 0.044) or chromatin heterogeneous (CHE, < 0.044).

### Stroma fraction

Stroma fraction was automatically calculated from the digital scan of the H&E-stained sections and stroma analysis software as previously reported [23]. Images of H&E-stained histological sections were scanned with the 40× lens on an Aperio AT2 scanner (Leica, Germany). The tumor areas were delineated on the scanned images by a pathologist with software (Room 4, Kent, UK). Tumors with a stroma fraction less than or equal to 50% were annotated as having a low stroma content, while tumors with a stroma fraction greater than 50% were annotated as having a high stroma content (Additional file 1: Fig. S1).

### Follow-up

Patients were monitored at 3-month intervals for the first 2 years and then biannually for 5 years after liver resection. Clinical examinations and CEA and carbohydrate antigen 19-9 (CA19-9) detection were performed every 3 months. Chest/abdominal/pelvic computed tomography (CT) and colonoscopy were performed annually. Recurrence-free survival (RFS) was defined as the interval from the date of liver metastasis resection to the date of disease recurrence, death, or the last follow-up. OS was defined as the interval from the date of liver metastasis resection to the date of death from any cause or to the last follow-up. Random censoring was applied to patients without recurrence or death at the last follow-up date. The final follow-up visit occurred in October 2020.

### Statistical analysis

Statistical analyses were performed using SPSS 24.0 software (IBM, Chicago, IL, USA) and R software packages (version 3.5.1). Categorical variables are presented as percentages and were compared using the chi-square test or Fisher’s exact test. TBS was calculated from the distance from the origin on a Cartesian plane incorporating the maximum tumor size (x-axis) and number of lesions (y-axis) [13]. Kaplan–Meier survival curves with log-rank estimates were used to depict time-to-event parameters. Multivariate Cox proportional hazards analysis was performed using variables whose *P* value was less than 0.05 in the univariate analysis. Hazard ratios (HRs) and 95% confidence intervals (CIs) were subsequently calculated. Wilcoxon matched-pair signed-rank tests were applied in the overall comparison of time-dependent area under curve (AUC) between groups. The survival curve was plotted with the survminer package (version 0.4.4; CRAN.R-project.org/package= survminer). Time-dependent AUC was calculated with the time ROC package (version 0.3; CRAN.R-project.org/package=timeROC). A two-sided *P* < 0.05 was considered statistically significant.

## Results

### Patient demographics

**Fig. 1:**
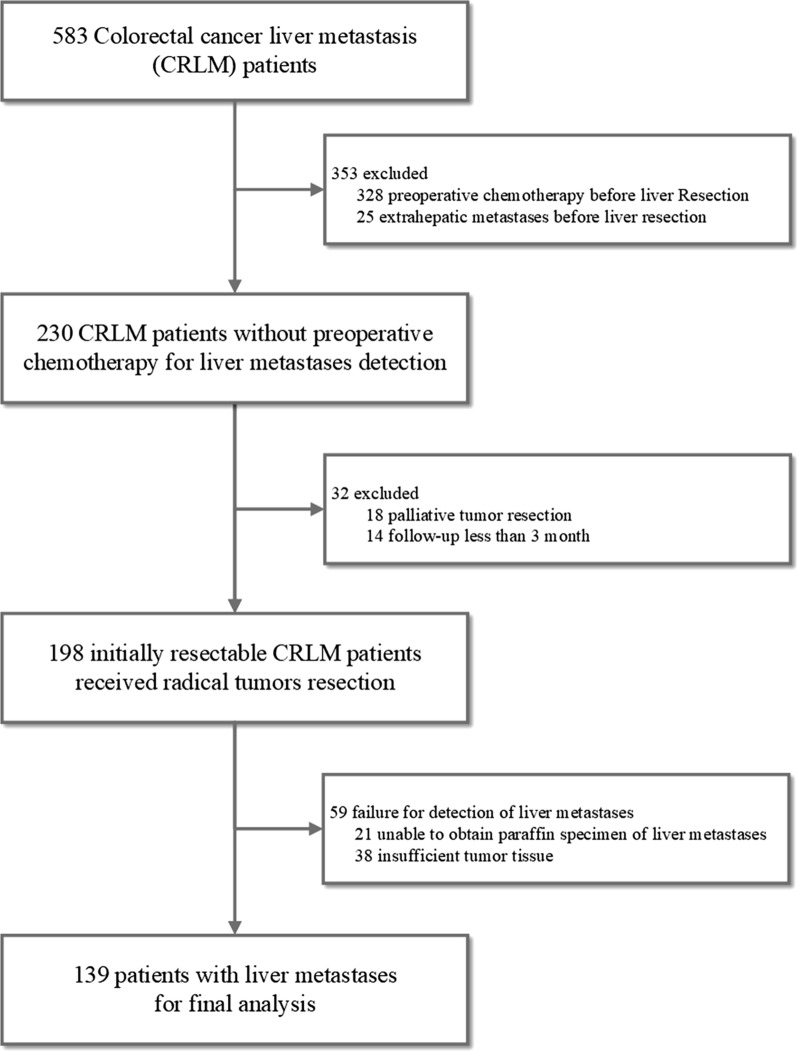
Flow chart of the total patient selection process

Four hundred forty-four of the 583 patients were excluded. The flowchart of the selection process is shown in Fig. 1. As a result, 139 patients were selected for this study. The clinical and pathological characteristics of the patients are presented in Table 1. The median age of all patients was 59.5 years (range 25–84 years), and 61.9% of the patients were male. Eighty-five (61.2%) patients received adjuvant chemotherapy after liver resection. Among them, 41 (48.2%) patients received XELOX regimen (Oxaliplatin 130 mg/m^2^ intravenous injection, day 1, Capecitabine 1000 mg/m^2^, orally twice a day, day 1–14), 35 (41.2%) patients received mFOLFOX6 regimen [Oxaliplatin 85 mg/m^2^ intravenous infusion, day 1, Leucovorin 400 mg/m^2^ intravenous infusion, day 1, 5-Fluorouracil 400 mg/m^2^ intravenous bolus injection, day 1, then 1200 mg/m^2^/d×2 days continuous intravenous infusion (Total 2400 mg/m^2^, 46~48 h infusion)], and 9 (10.6%) patients received single-agent capecitabine regimen (Capecitabine 850-1250 mg/m^2^ orally, twice a day, day 1–14). After a median follow-up time of 40 months (25–75% quartiles: 28–57 months), 80 (57.6%) patients were alive with a tumor-free status, 21 (15.1%) patients were alive with tumor recurrence, including 11 (52.4%) liver tumor recurrence, 4 pulmonary metastases, 3 peritoneal metastases, 1 liver and pulmonary metastasis and 1 liver and bone metastasis. And 38 (27.3) patients experienced cancer-related mortality. The 3-year RFS rate and OS rate were 65.0% and 84.2%, respectively.

**Table 1:** Characteristics of included patients and analysis of liver metastasis tumors

Parameters	Total patients (n, %)
Median age (years)	59.5 (25-84)
Age, years	
≤ 60	75 (54.0)
> 60	64 (46.0)
Sex	
Male	86 (61.9)
Female	53 (38.1)
Primary tumor location	
Colon	97 (69.8)
Rectum	42 (30.2)
Primary tumor differentiation	
Well to moderate	108 (77.7)
Poor	31 (22.3)
T stage	
T1–3	96 (69.1)
T4	43 (30.9)
N stage	
N0	50 (36.0)
N1–2	89 (64.0)
Timing of liver metastases	
Synchronous	75 (54.0)
Metachronous	64(46.0)
Number of liver metastases	
1	91 (65.5)
2	29 (20.9)
3	12(8.6)
4	4 (2.9)
5	3 (2.2)
Liver metastases diameter (cm)	
Median (range)	2.5 (0.5-8.4)
≤ 3	99 (71.2)
> 3	40 (28.8)
Distribution of liver metastases	
Unilobar	117 (84.2)
Bilobar	22 (15.8)
TBS	
Median (range)	2.83 (1.12-8.46)
≤ 3	77 (55.4)
> 3	62 (44.6)
Median liver resection margin (cm)	0.8 (0-3.5)
Adjuvant chemotherapy after liver resection	
Yes	85 (61.2)
No	54 (38.8)
DNA ploidy	
Diploid	32 (23.0)
Aneuploid	95 (68.3)
Tetraploid	12 (8.6)
Stroma fraction	
Low stroma	85 (61.2)
High stroma	54 (38.8)
Nucleotyping	
Chromatin homogeneous	79 (56.8)
Chromatin heterogeneous	60 (43.2)

*TBS* tumor burden score

### DNA ploidy, stroma fraction and nucleotyping

The diploid DNA ploidy was classified in liver metastases from 32 patients (23%), whereas 107 (77.0%) patients were classified as nondiploid. A low stroma fraction of liver metastases was observed in 85 (61.2%) patients, and a high stroma fraction was found in 54 (38.8%) patients. Regarding the nucleotyping of liver metastases, chromatin homogeneity was found in 79 (56.8 %) patients, while chromatin heterogeneity was found in 60 (43.2%) patients. No significant association was observed between DNA ploidy, stroma fraction and nucleotyping of liver metastases and clinicopathological characteristics (Additional file 2: Table S1).

### Survival analyses

**Fig. 2:**
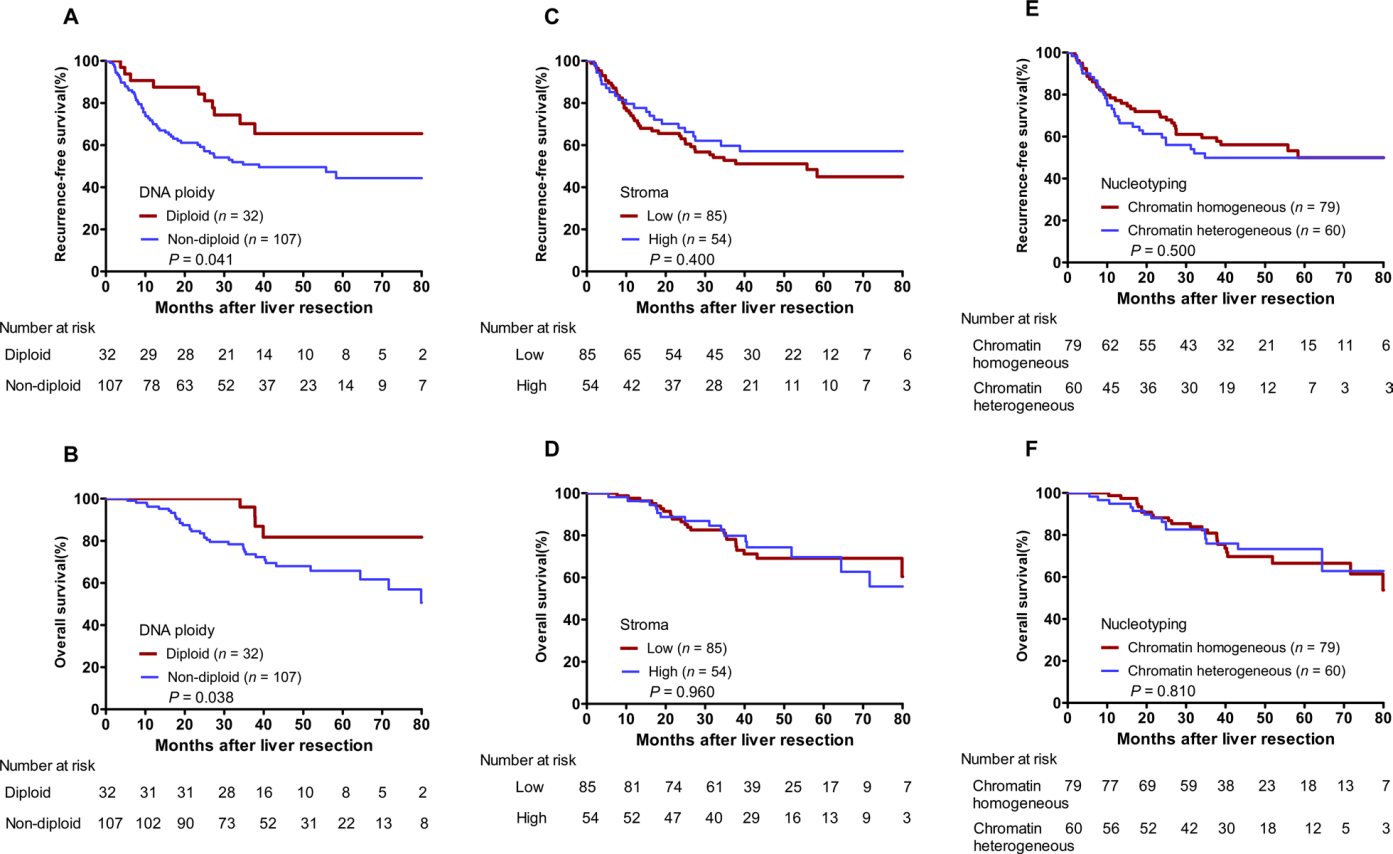
Kaplan-Meier curves of patients with initially resectable CRLM grouped by DNA ploidy, stroma fraction and nucleotyping. **A** Comparison of recurrence-free survival (RFS) between the diploid DNA ploidy group and the nondiploid DNA ploidy group. **B** Comparison of overall survival (OS) between the diploid DNA ploidy group and the nondiploid DNA ploidy group. **C** Comparison of RFS between the high stroma fraction group and the low stroma fraction group. **D** Comparison of OS between the high stroma fraction group and the low stroma fraction group. **E** Comparison of RFS between the chromatin heterogeneous group and the chromatin homogeneous group. **F** Comparison of OS between the chromatin heterogeneous group and the chromatin homogeneous group

**Fig. 3:**
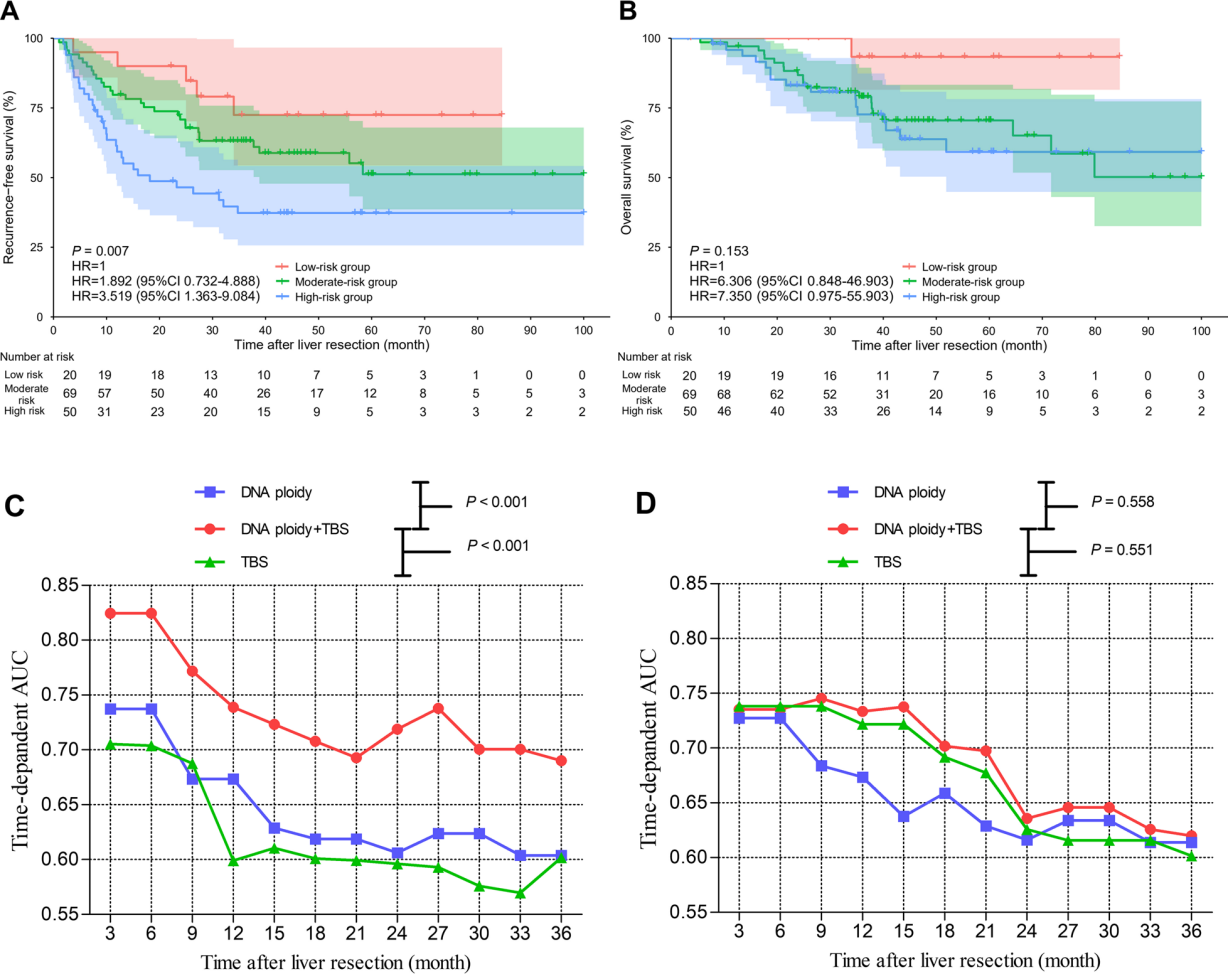
**A** Comparison of recurrence-free survival of patients with initially resectable CRLM stratified by DNA ploidy and tumor burden score (TBS) from the low-risk (diploid DNA ploidy and TBS ≤ 3), moderate-risk (diploid DNA ploidy and TBS > 3 or nondiploid DNA ploidy and TBS ≤ 3) and high-risk (nondiploid DNA ploidy and TBS > 3) groups. **B** Comparison of overall survival of patients with initially resectable CRLM stratified by DNA ploidy and TBS from the low-risk, moderate-risk and high-risk groups. **C** Recurrence-free survival related time-dependent areas under curve (AUCs) of the DNA ploidy, TBS and DNA ploidy plus TBS. **D** Overall survival related time-dependent AUCs of the DNA ploidy, TBS and DNA ploidy plus TBS

Patients with nondiploid DNA ploidy had a worse 3-year RFS rate (50.8% vs. 70.1%, *P* = 0.041, Fig. 2A) and a worse 3-year OS rate (73.6% vs. 96.0%, *P* = 0.038, Fig. 2B) than those with diploid DNA ploidy. Patients with either a high or low stroma fraction presented comparable 3-year RFS rate (59.7% vs. 52.7%, *P* = 0.400, Fig. 2C) and 3-year OS rate (79.8% vs. 78.1%, *P* = 0.960, Fig. 2D). Similarly, patients with either chromatin heterogeneous or chromatin homogeneous liver metastases showed comparable 3-year RFS rate (49.8% vs. 59.4%, *P* = 0.500, Fig. 2E) and 3-year OS rate (75.9% vs. 80.8%, *P* = 0.810, Fig. 2F).

The results of univariate and multivariate analyses of RFS are summarized in Table 2. The univariate analysis revealed that the N1-2 stage, TBS > 3, and nondiploid DNA ploidy were associated with unfavorable RFS. The multivariate analysis showed that the N1-2 stage (HR, 3.260; 95% CI 1.765–6.023; *P* < 0.001), TBS > 3 (HR, 1.817; 95% CI 1.106–2.984; *P* = 0.018), nondiploid DNA ploidy (HR, 2.082; 95% CI 1.053–4.115; *P* = 0.035) were also independent predictive factors for an unfavorable RFS. The results of univariate and multivariate analyses of OS are summarized in Table 3. The univariate analysis revealed that rectal cancer, T4 stage, N1-2 stage, synchronous liver metastases, TBS > 3, and nondiploid DNA ploidy were associated with unfavorable OS. The multivariate analysis showed that the N1-2 stage (HR, 3.421; 95% CI 1.493–7.841; *P* = 0.004) and rectal cancer (HR, 2.777; 95% CI 1.371–5.626; *P* = 0.005) were independent predictive factors for unfavorable OS. The results showed that the nondiploid DNA ploidy of liver metastases and TBS are negative prognostic factors for patients with initially resectable CRLM after liver resection, so we combined both of them as a novel parameter in the following study.

**Table 2:** Univariate and multivariate analyses of risk factors influencing RFS after liver resection

Variable	Univariate		Multivariate	
	HR (95 % CI)	*P* value	HR (95 % CI)	*P* value
Age (> 60 years vs. ≤ 60 years)	0.677 (0.408-1.123)	0.131		
Sex (Male vs. Female)	0.961 (0.583-1.585)	0.877		
Primary tumor location (Rectum vs. Colon)	1.373 (0.824-2.290)	0.224		
Primary tumor differentiation (Poor vs. Well to moderate)	1.246 (0.707-2.194)	0.447		
T stage (T4 vs. T1–3)	1.234 (0.731-2.082)	0.431		
N stage (N1–2 vs. N0)	3.067 (1.663-5.656)	*< 0.001*	3.260 (1.765-6.023)	*< 0.001*
Timing of liver metastasis (Synchronous vs. Metachronous)	0.796 (0.487-1.302)	0.364		
TBS (>3 vs. ≤3)	1.863(1.137-3.050)	*0.013*	1.817 (1.106-2.984)	*0.018*
Adjuvant chemotherapy after liver resection (Yes vs. No)	1.292 (0.748-2.231)	0.359		
DNA ploidy (Nondiploid vs. Diploid)	1.997 (1.016-3.925)	*0.045*	2.082 (1.053-4.115)	*0.035*
Stroma fraction (High stroma vs. Low stroma)	0.801 (0.478-1.343)	0.400		
Nucleotyping (Chromatin heterogeneous vs. Chromatin homogeneous)	1.184 (0.724-1.939)	0.501		

Italic represents when the p-value result is less than 0.05

*RFS *recurrence-free survival*, HR *hazard ratio, *CI *confidence interval, *TBS *tumor burden score

**Table 3:** Univariate and multivariate analyses of risk factors influencing OS after liver resection

Variable	Univariate		Multivariate	
	HR (95 % CI)	*P* value	HR (95% CI)	*P* value
Age (> 60 years vs. ≤ 60 years)	0.745 (0.384−1.443)	0.382		
Sex (Male vs. Female)	1.056 (0.550−2.027)	0.870		
Primary tumor location (Rectum vs. Colon)	2.565 (1.355−4.854)	*0.004*	2.777 (1.371−5.626)	*0.005*
Primary tumor differentiation (Poor vs. Well to moderate)	1.000 (0.458−2.182)	0.999		
T stage (T4 vs. T1-3)	2.185 (1.145-4.169)	*0.018*	1.241 (0.598-2.576)	0.562
N stage (N1−2 vs. N0)	3.067 (1.663−5.656)	*< 0.001*	3.421 (1.493−7.841)	*0.004*
Timing of liver metastasis (Synchronous vs. Metachronous)	2.955 (1.346−6.488)	*0.007*	0.957 (0.465−1.967)	0.904
TBS (>3 vs. ≤3)	1.315 (0.696−2.485)	0.399		
Adjuvant chemotherapy after liver resection (Yes vs. No)	0.845 (0.423−1.689)	0.634		
DNA ploidy (Nondiploid vs. diploid)	2.855 (1.013−8.049)	*0.047*	2.751 (0.971-7.795)	0.057
Stroma fraction (High stroma vs. low stroma)	0.984 (0.513−1.889)	0.962		
Nucleotyping (Chromatin heterogeneous vs. Chromatin homogeneous)	0.924 (0.481−1.774)	0.812		

Italic represents when the p-value result is less than 0.05

*RFS *recurrence-free survival, *HR *hazard ratio,* CI *confidence interval,* TBS *tumor burden score

### Identification of risk groups

The combination of DNA ploidy and TBS were also analyzed. With respect to RFS, the combination of DNA ploidy and TBS was divided into three risk groups. Patients with diploid DNA ploidy and TBS ≤ 3 (low-risk group) had the highest 3-year RFS rate [72.5% (95% CI 45.5–87.7%)] of the three groups. Patients with diploid DNA ploidy of liver metastases and TBS > 3 or nondiploid DNA ploidy and TBS ≤ 3 (moderate-risk group) presented intermediate 3-year RFS rates [63.2% (95% CI73.4–50.5%)], and the HR was 1.892 (95% CI 0.732–4.888). Patients with nondiploid DNA ploidy and TBS > 3 (high-risk group) had the lowest 3-year RFS rate [37.3% (95% CI 23.7–50.9%)], and the HR was 3.519 (95% CI 1.363–9.084). The combination of DNA ploidy and TBS was statistically significant for RFS (*P* = 0.007) (Fig. 3A), while the combination of DNA ploidy and TBS was not statistically significant for OS (*P* = 0.153) (Fig. 3B). The recurrence-free survival related time-dependent AUCs of DNA ploidy plus TBS were significantly larger than those of DNA ploidy and TBS at a series of time points (Fig. 3C). The overall survival related time-dependent AUCs of DNA ploidy plus TBS, DNA ploidy and TBS at a series of time points were not of significant difference (Fig. 3D). Combination ofDNA ploidy and TBS was proven to stratify patients with initially resectable CRLM into different risk groups.

**Fig. 4:**
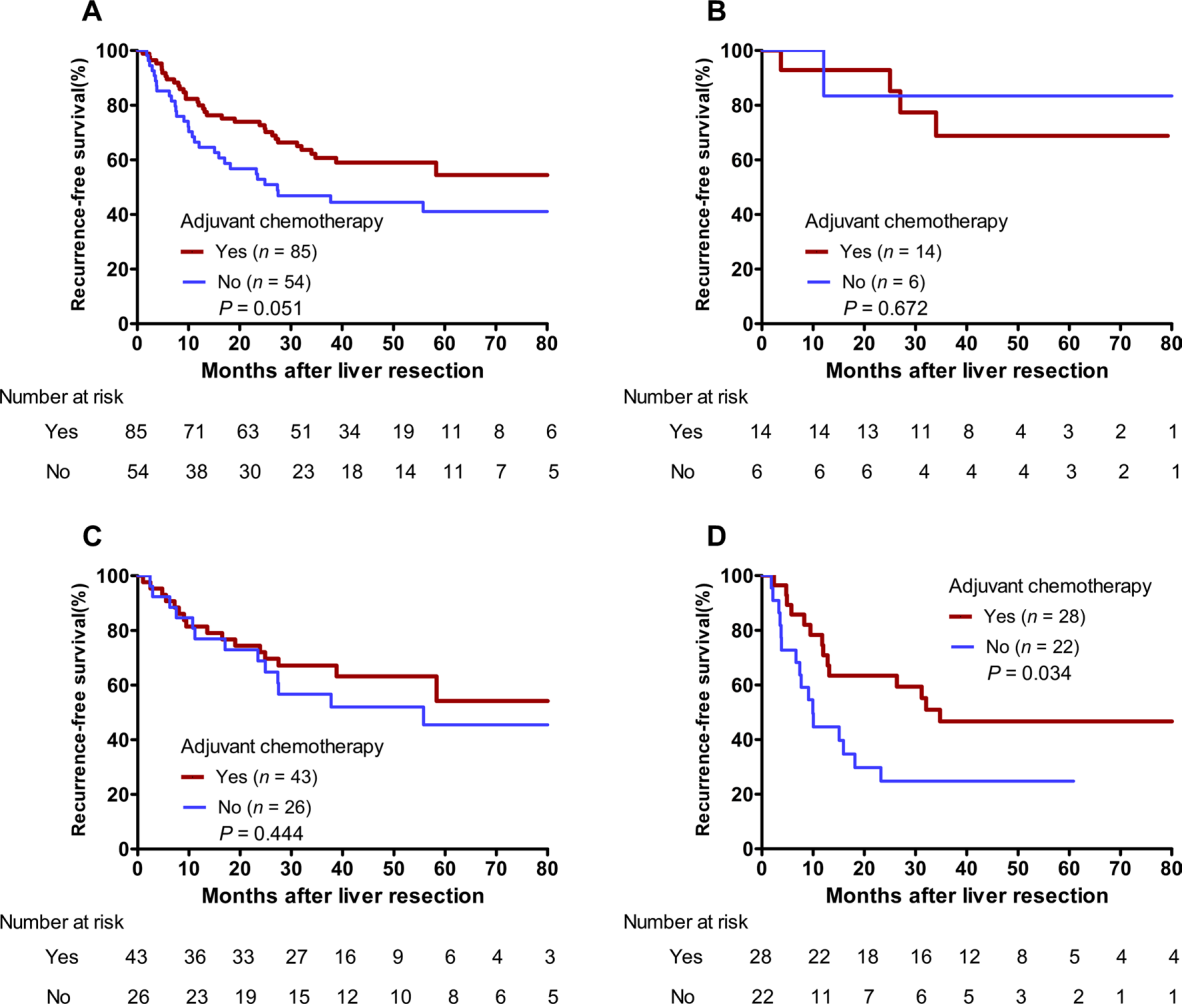
Kaplan-Meier curves of recurrence-free survival of patients with initially resectable CRLM with and without adjuvant chemotherapy stratified by different risk groups. **A** Total patients. **B** low-risk group (diploid DNA ploidy and TBS ≤ 3). **C** moderate-risk group (diploid DNA ploidy and TBS > 3 or nondiploid DNA ploidy and TBS ≤ 3). **D** high-risk group (nondiploid DNA ploidy and TBS > 3)

Comparisons of the 3-year RFS rates between the patients with and without adjuvant chemotherapy stratified by different risk groups are shown in Fig. 4. There were no significant difference of RFS rate in total patients as well as in low-risk group and moderate-risk group (Total patients: 60.7% vs. 46.8%; *P* = 0.051; Fig. 4A; low-risk: 68.8% vs. 83.3%; *P* = 0.672; Fig. 4B; moderate-risk: 67.1% vs. 56.7%; *P* = 0.444; Fig. 4C). However, the high-risk group who received adjuvant chemotherapy had a significantly better 3-year RFS rate (46.7% vs. 24.8%; *P* = 0.034) (Fig. 4D) than those without adjuvant chemotherapy. For the high-risk patients, they could benefit from the adjuvant chemotherapy after liver resection.

## Discussion

The clear prognostic prediction for patients with CRLM requires precise measurements of the gross tumor and cellular structures of liver metastases. In the current study, we first investigated tumor cell structure characteristics, including DNA ploidy, stroma fraction and nucleotyping in liver metastases from patients with CRLM who underwent liver resection. Our data analyses showed that patients with nondiploid DNA ploidy of liver metastases had worse 3-year RFS and OS rates than patients with diploid DNA ploidy. However, stroma fraction and nucleotyping did not show a significant prognostic prediction effect on the patients with CRLM. Subsequently, we constructed a novel parameter, the combination of DNA ploidy and TBS, which was proven to be capable of stratifying patients with CRLM into low-, moderate- and high-risk groups with 3-year RFS rates of 72.5%, 63.2% and 37.3%, respectively.

Nondiploid DNA serves as a negative prognostic factor for patients with nonmetastatic CRC in previous studies [23, 29]. In patients with metastatic CRC, a high DNA ploidy score was associated with a higher probability of death [30]. Similarly, the nondiploid DNA ploidy of liver metastases was also proven to be a negative prognostic factor of RFS in the present study. The mechanistic link between nondiploid DNA ploidy and a poor prognosis might be because nondiploid DNA ploidy was proven to be related to an aggressive tumor behavior [31] and a poor chemotherapy response [32, 33]. Previous studies have provided evidence supporting the hypothesis that both stroma fraction and nucleotyping are significant prognostic factors for early-stage CRC [21, 22, 29]. In contrast to previous studies, our results showed that neither stroma fraction nor nucleotyping was associated with postoperative survival in patients with CRLM. This may be due to the heterogeneity of different liver metastases from the same patients. The detection of one of multiple liver metastases might not sufficient to obtain complete information on stroma fraction and nucleotyping classification. In addition, the prognostic value of stroma fraction and nucleotyping was proven based on the result from primary CRC tumors in previous studies [21, 22, 25, 26], and our results showed that they may not be applicable to CRC patients with liver metastases.

TBS was validated to be an accurate tool to account for the effect of tumor morphology on long-term survival among patients with CRLM who were undergoing resection, with excellent prognostic discriminatory power 13, 34. Patients with a high TBS of CRLM tended to have a higher R1 resection rate, indicating a higher possibility of postoperative recurrence in patients who significantly benefitted from receiving systemic chemotherapy 14. Our study innovatively combined TBS and DNA ploidy as a novel parameter that provided a better stratification of patients into low-, moderate- and high-risk groups for 3-year RFS. There are several advantages in combining TBS and DNA ploidy as a novel parameter which needs to express. Firstly, combining TBS and DNA ploidy allows complete assessment on essential pathological features including tumor morphology and cell numbers and chromosome instability respectively. In addition, our research results showed that the combined parameter was better in predicting RFS than single TBS or DNA ploidy, suggesting that it has an excellent prognostic value in predicting postoperative recurrence after liver resection. Moreover, these two biomarkers, TBS and DNA ploidy, are convenient to measure, which is suitable for clinical practice.

Based on the survival results of adjuvant chemotherapy stratified by different risk groups, TBS and DNA ploidy was able to help guiding the postoperative treatment among the patients with CRLM. For the high-risk patients, they could benefit from the adjuvant chemotherapy, thus they should receive a sufficient duration or aggressive postoperative treatment. For the moderate-risk group and low-risk group, whether to conduct adjuvant chemotherapy remains controversial. Accumulating evidence supports the hypothesis that postoperative chemotherapy fails to prolong the survival of patients with CRLM presenting with a low risk of recurrence [8, 35, 36]. As no survival benefit of adjuvant chemotherapy was observed in moderate-risk group and low-risk group of CRLM patients in the current study, we suggested that routine follow-up might be enough for the moderate-risk group and low-risk group. And for moderate-risk group, the difference is close to statistical difference, if number of patients was larger, result may be different. As a result, for moderate-risk group, chemotherapy decision requires other consideration factors such as age, general health.

Several limitations to the current study should be acknowledged. First, this retrospective study included an uncontrolled methodology and a limited number of patients recruited from a single cohort. Selective bias exists, and the findings must be validated in external cohorts. Second, the 5-year survival data were unavailable for some patients due to an insufficient follow-up duration. This issue may have led to the underestimation or overestimation of the prognostic effect of DNA ploidy, stroma fraction and nucleotyping on liver metastases. Additionally, several tumor molecular markers were not included in the current study. RAS, BRAF,TP53, and SMAD4 mutations are significantly associated with the long-term survival of patients with CRLM after liver resection [37, 38]. A confirmation of the association of DNA ploidy in liver metastases with potential driver gene mutations would help us further understand the effect of DNA ploidy on the postoperative recurrence of CRLM.

## Conclusions

As shown in the present study, the nondiploid DNA ploidy of liver metastases is a negative prognostic factor for patients with initially resectable CRLM after liver resection. A novel parameter that combined DNA ploidy and TBS was proven to stratify patients with initially resectable CRLM into different risk groups and may guide individual postoperative treatment for patients with initially resectable CRLM.

## Supplementary Information

**Additional file 1: Figure S1.** Representative images of H&E-stained histological sections. (A) Image of low stroma 1 (25%); (B) Image of low stroma 2 (31%); (C) Image of high stroma 1 (64%); (D) Image of high stroma 2 (76%).

**Additional file 2: Table S1.** The association of the DNA ploidy, stroma fraction and nucleotyping ofliver metastases and the clinicopathological features.

## Data Availability

The datasets used and analyzed during the current study are available from the corresponding author on reasonable request. The authenticity of this article has been validated by uploading the key raw data onto the Research Data Deposit public platform (www. researchdata.org.cn).

## Declarations

### Data availability statement

Data availability statement the datasets used and analyzed during the current study are available from the corresponding author on reasonable request. The authenticity of this article has been validated by uploading the key raw data onto the Research Data Deposit public platform (http://www.researchdata.org.cn), with the Approval Number as RDDA2021233167.
