# Supplementary figures and images for: Prognostic value of a novel biomarker combining DNA ploidy and tumor burden score for initially resectable liver metastases from patients with colorectal cancer
Source: Cancer Cell Int. 2021 Oct 23;21:554. doi: 10.1186/s12935-021-02250-x (PMC8542290; doi:10.1186/s12935-021-02250-x)

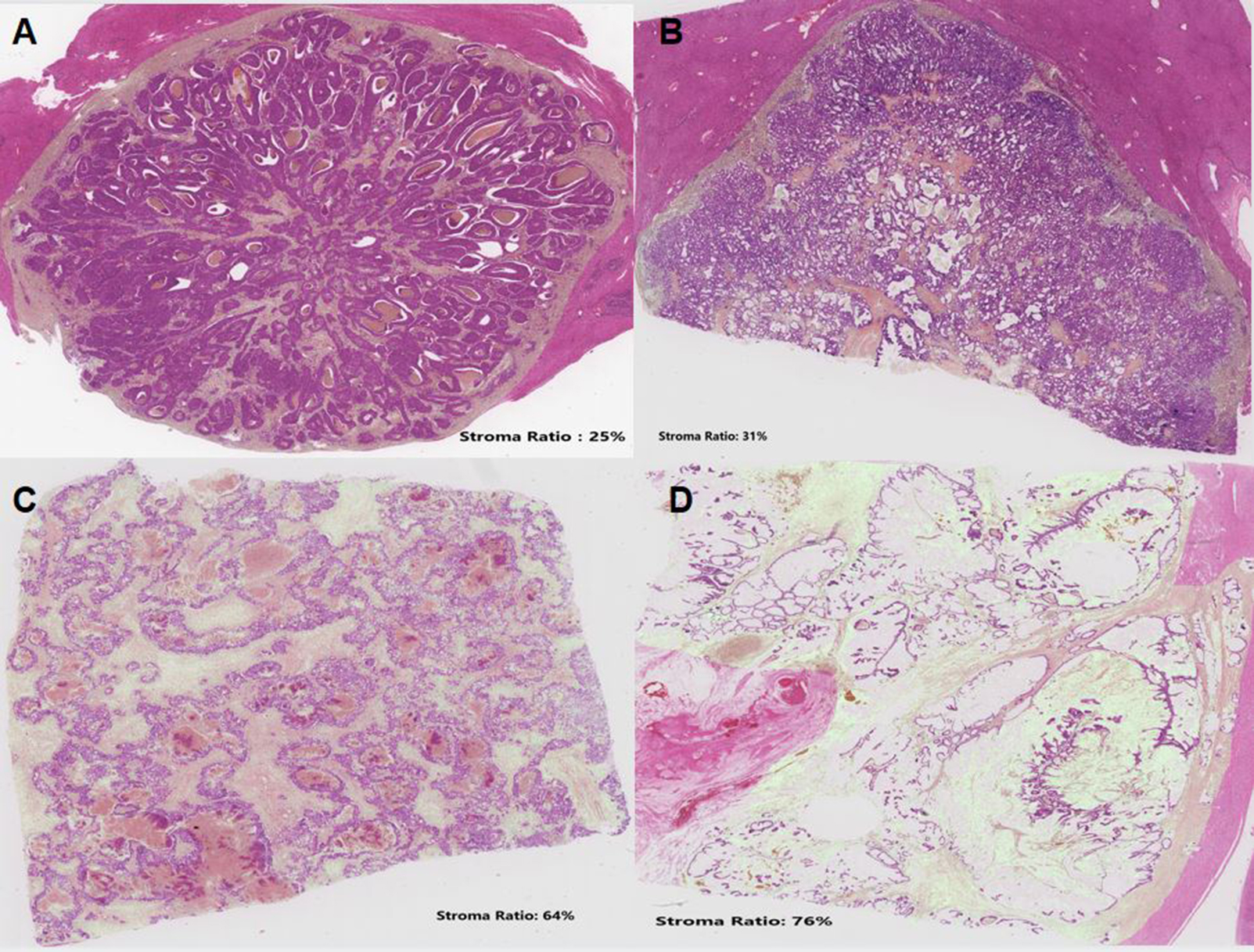

Supplement: Supplementary file 1 — Additional file 1: Figure S1. Representative images of H&E-stained histological sections. (A) Image of low stroma 1 (25%); (B) Image of low stroma 2 (31%); (C) Image of high stroma 1 (64%); (D) Image of high stroma 2 (76%). [file 12935_2021_2250_MOESM1_ESM.jpg]
