# Supplementary material for: Prognostic value of a novel biomarker combining DNA ploidy and tumor burden score for initially resectable liver metastases from patients with colorectal cancer
Source: Cancer Cell Int. 2021 Oct 23;21:554. doi: 10.1186/s12935-021-02250-x (PMC8542290; doi:10.1186/s12935-021-02250-x)
Supplement: Supplementary file 2 — Additional file 2: Table S1. The association of the DNA ploidy, stroma fraction and nucleotyping ofliver metastases and the clinicopathological features. [file 12935_2021_2250_MOESM2_ESM.docx]

**Table S1. The association of the DNA ploidy, stroma fraction and nucleotyping of liver metastases and the clinicopathological features.**

| **Parameters** | **DNA ploidy** | | |  | **Stroma fraction** | | |  | **Nucleotyping** | | |
| --- | --- | --- | --- | --- | --- | --- | --- | --- | --- | --- | --- |
|  | Diploid | Non-diploid | *P value* |  | Low stroma | High stroma | *P value* |  | Chromatin homogeneous | Chromatin heterogeneous | *P value* |
| Sex |  |  | 0.618 |  |  |  | 0.569 |  |  |  | 0.966 |
| Male | 21 (65.6) | 65 (60.7) |  |  | 51 (60.0) | 35 (64.8) |  |  | 49 (62.0) | 37 (61.7) |  |
| Female | 11 (34.4) | 42 (39.3) |  |  | 34 (40.0) | 19 (35.2) |  |  | 30 (38.0) | 23 (38.3) |  |
| Age |  |  | 0.360 |  |  |  | 0.763 |  |  |  | 0.213 |
| ≤60 | 15 (46.9) | 60 (56.1) |  |  | 45 (52.9) | 30 (55.6) |  |  | 39 (49.4) | 36 (60.0) |  |
| >60 | 17 (53.1) | 47 (43.9) |  |  | 40 (47.1) | 24 (44.4) |  |  | 40 (50.6) | 24 (40.0) |  |
| Primary tumor location |  |  | 0.769 |  |  |  | 0.618 |  |  |  | 0.427 |
| Colon | 23 (71.9) | 74 (69.2) |  |  | 58 (68.2) | 39 (72.2) |  |  | 53 (67.1) | 44 (73.3) |  |
| Rectum | 9 (28.1) | 33 (30.8) |  |  | 27 (31.8) | 15 (27.8) |  |  | 26 (32.9) | 16 (26.7) |  |
| Primary tumor differentiation |  |  | 0.676 |  |  |  | 0.393 |  |  |  | 0.875 |
| Well to moderate | 24 (75.0) | 84 (78.5) |  |  | 64 (75.3) | 44 (81.5) |  |  | 61 (77.2) | 47 (78.3) |  |
| Poor | 8 (25.0) | 23 (21.5) |  |  | 21 (24.7) | 10 (18.5) |  |  | 18 (22.8) | 13 (21.7) |  |
| T stage |  |  | 0.695 |  |  |  | 0.626 |  |  |  | 0.100 |
| 1-3 | 23 (71.9) | 73 (68.2) |  |  | 60 (70.6) | 36 (66.7) |  |  | 59 (74.7) | 37 (61.7) |  |
| 4 | 9 (28.1) | 34 (31.8) |  |  | 25 (29.4) | 18 (33.3) |  |  | 20 (25.3) | 23 (38.3) |  |
| N stage |  |  | 0.830 |  |  |  | 0.605 |  |  |  | 0.357 |
| 0 | 11 (34.4) | 39 (36.4) |  |  | 32 (37.6) | 18 (33.3) |  |  | 31 (39.2) | 19 (31.7) |  |
| 1-2 | 21 (65.6) | 68 (63.6) |  |  | 53 (62.4) | 36 (66.7) |  |  | 48 (60.8) | 41 (68.3) |  |
| Timing of liver metastases |  |  | 0.767 |  |  |  | 0.763 |  |  |  | 0.898 |
| Synchronous | 18 (56.3) | 57 (53.3) |  |  | 45 (52.9) | 30 (55.6) |  |  | 43 (54.4) | 32 (53.3) |  |
| Metachronous | 14 (43.8) | 50 (46.7) |  |  | 40 (47.5) | 24 (44.4) |  |  | 36 (45.6) | 28 (46.7) |  |
| Liver metastases diameter (cm) |  |  | 0.326 |  |  |  | 0.183 |  |  |  | 0.158 |
| ≤3 | 25 (78.1) | 74 (69.2) |  |  | 64 (75.3) | 35 (64.8) |  |  | 60 (75.9) | 39 (65.0) |  |
| >3 | 7 (21.9) | 33 (30.8) |  |  | 21 (24.7) | 19 (35.2) |  |  | 19 (24.1) | 21 (21.0) |  |
| Number of liver metastases |  |  | 0.656 |  |  |  | 0.547 |  |  |  | 0.237 |
| 1 | 22 (68.8) | 69 (64.5) |  |  | 54 (63.5) | 37 (68.5) |  |  | 55 (69.6) | 36 (60.0) |  |
| 2-5 | 10 (31.3) | 38 (35.5) |  |  | 31 (36.5) | 17 (31.5) |  |  | 24 (30.4) | 24 (40.0) |  |
| Liver metastases distribution |  |  | 0.254 |  |  |  | 0.794 |  |  |  | 0.24 |
| Unilobar | 29 (90.6) | 88 (82.2) |  |  | 71 (83.5) | 46 (85.2) |  |  | 69 (87.3) | 48 (80.0) |  |
| Bilobar | 3 (9.4) | 19 (17.8) |  |  | 14 (16.5) | 8 (14.8) |  |  | 10 (12.7) | 12 (20.0) |  |
| TBS |  |  | 0.357 |  |  |  | 0.503 |  |  |  | 0.265 |
| ≤3 | 20 (62.5) | 57 (53.3) |  |  | 49 (57.6) | 28 (51.9) |  |  | 47 (59.5) | 30 (50.0) |  |
| >3 | 12 (37.5) | 50 (46.7) |  |  | 36 (42.4) | 26 (48.1) |  |  | 32 (40.5) | 30 (50.0) |  |
| Adjuvant chemotherapy after liver resection |  |  | 0.656 |  |  |  | 0.389 |  |  |  | 0.920 |
| Yes | 22 (68.8) | 69 (35.5) |  |  | 58 (68.2) | 33 (61.1) |  |  | 52 (65.8) | 39 (65.0) |  |
| No | 10 (31.2) | 38 (64.5) |  |  | 27 (31.8) | 21 (38.9) |  |  | 27 (34.2) | 21 (35.0) |  |

*TBS, tumor burden score.*
